# Pilot-Scale Evaluation of Flat-Sheet Membrane Bioreactor for In Situ Retrofitting Textile Dyeing Wastewater Treatment Plant

**DOI:** 10.3390/membranes16020059

**Published:** 2026-02-02

**Authors:** Chaoqun Zhou, Chunhai Wei, Huarong Yu, Hongwei Rong, Kang Xiao

**Affiliations:** 1 Department of Municipal Engineering, School of Civil Engineering and Transportation, Guangzhou University, Guangzhou 510006, China; 13940404533@163.com (C.Z.); huarongyu@gzhu.edu.cn (H.Y.); hwrong@gzhu.edu.cn (H.R.); 2 Guangzhou Sewage Purification Co., Ltd., Guangzhou 510630, China; 3 Key Laboratory for Water Quality and Conservation of the Pearl River Delta, Ministry of Education, Guangzhou 510006, China; 4 Beijing Yanshan Earth Critical Zone National Research Station, College of Resources and Environment, University of Chinese Academy of Sciences, Beijing 101408, China; 5 State Key Laboratory of Earth System Numerical Modeling and Application, University of Chinese Academy of Sciences, Beijing 101408, China

**Keywords:** membrane fouling, chroma, turbidity, colloids, macromolecular organics, reverse osmosis pretreatment, wastewater reuse, contact angle, permeability, zeta potential

## Abstract

It is promising to in situ retrofit the activated sludge process with a membrane bioreactor (MBR) to increase treatment capacity and improve effluent quality in a textile dyeing wastewater treatment plant (WWTP). Membrane selection among commercial products for real engineering applications is critical for this specific wastewater, and little information is available in the literature. This study systematically evaluated the application potential of two flat-sheet microfiltration membranes made of polyvinylidene fluoride (PVDF) and polyether sulfone (PES) in pilot-scale MBRs for in situ retrofitting textile dyeing WWTP. During the four stages with different loads, both membranes achieved nearly the same effluent quality and rejection performance. Both membranes showed little trans-membrane pressure (TMP) increase at an average flux of 15 L/(m^2^·h) with sub-critical flux characteristics, and showed a sharp TMP increase with super-critical flux characteristics observed at an average flux of 18/22.5 L/(m^2^·h). After 74 d of filtration, at an average sludge concentration of 12,000 g/L, the PVDF membrane showed less variation in pore size distribution and bubble point pressure, while the PES membrane showed less change in permeability and contact angle. Both membranes met general MBR requirements due to the minimizing pristine effects of both membranes by this specific wastewater matrix. The PVDF membrane showed better anti-fouling capability, especially during high-/over-load stages, and thus was suggested for MBR retrofit, with a sustainable membrane flux below 18 L/(m^2^·h).

## 1. Introduction

China is the largest textile producer in the world. In 2024, the textile industry wastewater discharged a chemical oxygen demand (COD) of 34,189 tons and total nitrogen (TN) of 6072 tons, accounting for 17.9% and 13.8% of total industrial wastewater emissions in China, respectively [1]. The pollutants in the textile industry mainly originated from dyeing wastewater (approximately 80%) [2]. Dyeing wastewater shows high color intensity and thus obstructs light diffusion when directly discharging into water bodies. Furthermore, the residual dyes may generate toxic and harmful substances during migration and transformation processes, posing negative impacts on the ecological environment and human health [3,4]. Therefore, effective treatment is essential to mitigate these risks [5]. Textile dyeing wastewater contains dyes and auxiliaries with poor biodegradability, often rendering conventional secondary biological treatment processes (i.e., activated sludge or biofilm) incapable of achieving effluent stable compliance with discharge standards [6,7]. With the implementation of the Water Pollution Prevention and Control Action Plan (Water Ten Measures) in China, water conservation and emission reduction requirements for the textile dyeing industry have progressively intensified [8]. Therefore, adopting an enhanced secondary biological treatment (e.g., membrane bioreactor, MBR) plus an advanced physical–chemical treatment approach (e.g., reverse osmosis, RO) to achieve wastewater reuse represents an effective measure to alleviate water scarcity and mitigate water pollution within textile dyeing industrial parks [9,10,11].

MBR is a wastewater treatment and reuse process that integrates traditional activated sludge biodegradation with micro-/ultra-filtration membrane separation [12]. The MBR process structure typically consists of a biological reactor and a membrane tank. The biological reactor primarily degrades organic pollutants in the wastewater, while the micro-/ultra-filtration membranes with pore sizes typically ranging from 0.01 to 0.4 μm in the membrane tank can produce particle-free effluent from the sludge mixture. The high rejection efficiency of micro-/ultra-filtration membranes prevents sludge flocs loss, thus offering higher sludge concentration, stronger resistance to shock loads, reduced footprint, and better effluent quality in MBR than conventional activated sludge processes with gravitational sludge–water separation. Consequently, the MBR process is widely applied in municipal wastewater treatment and holds significant potential for industrial wastewater treatment (e.g., that of chemical, textile dyeing, and pharmaceutical wastewater) [13,14,15,16].

The modular design of micro-/ultra-filtration membranes facilitates their installation in aeration and/or sedimentation tanks, thus making MBR an ideal retrofit for the conventional activated sludge process, especially for municipal wastewater treatment plants (WWTPs) [17]. In industrial WWTPs, MBR is also a promising retrofit because its effluent can directly serve as the feed to the reverse osmosis (RO) process, producing high-quality reclaimed water in the nearby industries, including pharmaceuticals, power plants, and petrochemicals [18,19]. Bilici et al. [20] demonstrated that treating textile dyeing wastewater via MBR with ceramic membranes followed by RO achieved a COD and color removal of 89.1% and 95.6%, respectively, thus producing RO effluent for dyeing reuse. Feng et al. [21] conducted a study on treating textile dyeing wastewater using a combined Fenton and MBR process, which produced the final effluent from the MBR system meeting the reuse standards for municipal recycled water. Due to the complex textile dyeing wastewater quality derived from different textile production processes, membrane selection is critical when MBR retrofitting in a specific textile dyeing WWTP. However, there is little information available in the literature on this matter.

Motivated by membrane selection requirements for MBR retrofitting in a textile dyeing WWTP serving jeans production, this study conducted parallel pilot-scale MBR experiments to evaluate the pollutant rejection efficiency, fouling development, and characteristic changes in two commercial flat-sheet microfiltration membranes made from different materials for textile dyeing wastewater treatment. The outcome of this study provided the technical support for membrane selection, revealed the appropriate operational parameters, and demonstrated the technical feasibility of the MBR process for specific textile dyeing wastewater treatment.

## 2. Materials and Methods

### 2.1. Brief Information on MBR Retrofit in Textile Dyeing WWTP

A textile dyeing WWTP located in Foshan, Guangdong Province, China, was selected. It received an integrated wastewater flow of 50,000 m^3^/d, discharged from the nearby textile dyeing industrial zone mainly for jeans production, with high-strength pollutants (e.g., pH of 11–14, total COD of 1000–1500 mg/L, soluble chromaticity of 400–500 dilution times, suspended solids of 400–600 mg/L, and sulfide of 20–50 mg/L). Furthermore, it employed the combined process of acid neutralization, coagulation, primary sedimentation, conventional activated sludge, secondary sedimentation, ozonation, and constructed wetland to make the effluent meet the discharge standards for the textile industry in China (GB 4287-2012) [22]. With the further increase of textile production in the industrial zone, this WWTP is expected to receive an additional wastewater increment of 25,000 m^3^/d and supply reuse water (30,000 m^3^/d) to the industrial zone, according to the governmental regulation. Limited by the land availability in this WWTP, the MBR retrofit comprising conventional activated sludge and additional RO was chosen for this upgrading project. Considering the specific wastewater characteristics, particularly that it contained a lot of textile fiber debris, it was decided to conduct the pilot-scale MBR comparative tests for membrane selection with two commercial flat-sheet microfiltration membranes, which would avoid the common fiber clogging problems that occur in hollow fiber membranes.

### 2.2. Pilot-Scale MBR Set-Up and Operation Parameters

**Figure 1:**
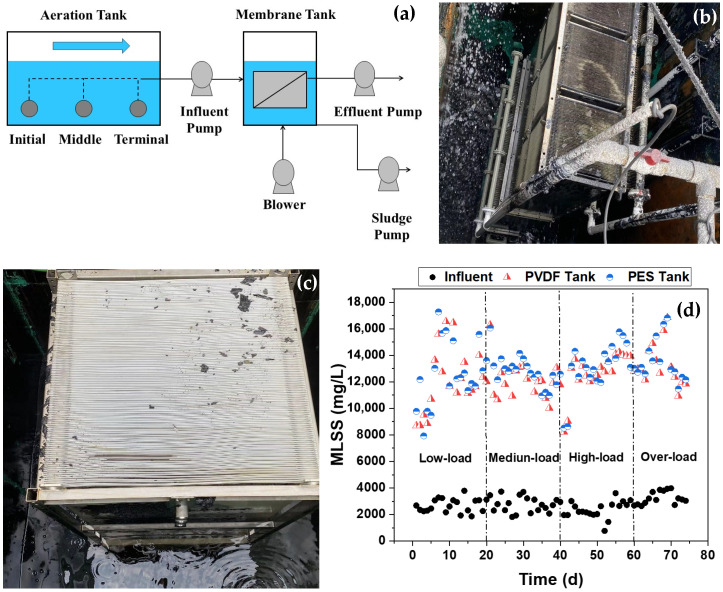
Pilot-scale MBR scheme (**a**), PVDF (**b**) and PES (**c**) flat-sheet membrane module, and MLSS in both membrane tanks (**d**).

Two pilot-scale MBRs were installed in the textile dyeing WWTP with the scheme shown in Figure 1a. Two commercial flat-sheet microfiltration membranes made of polyvinylidene fluoride (PVDF) and polyether sulfone (PES) were chosen for MBR evaluation. The PVDF membrane, with an effective area of 420 m^2^, and the PES membrane, with an effective area of 470 m^2^, were individually installed in stainless-steel rectangular tanks (with a field photo shown in Figure 1b and Figure 1c, respectively), close to the aeration tank in the textile dyeing WWTP. For each membrane tank, there were three pumps for delivering activated sludge mixed liquor in the aeration tank as influent, discharging concentrated sludge, and suctioning the membrane module for permeation. The suction pump was controlled by a timer with an on–off ratio of 8:2 min and was equipped with both a solenoid valve and a flowmeter for constant flow permeation. A pressure sensor in the membrane permeation pipe was employed to monitor the real-time pressure changes for calculating trans-membrane pressure (TMP). The continuous flows of the influent and discharging pumps were adjusted daily to maintain the sludge concentration at the target value of 12,000 mg/L in terms of mixed liquor suspended solids (MLSSs). Although there was a real MLSS fluctuation of 8000–17,000 mg/L in both membrane tanks, mainly due to the fluctuation of the influent MLSS, the MLSS in both membrane tanks was around 12,000 mg/L during most of the operation time, with nearly the same MLSS in both membrane tanks as observed in a side-by-side comparison between two membranes (shown in Figure 1d). Continuous air scouring along the membrane surface was provided at the ratio of air to permeate of 10:1 (i.e., specific aeration demand of 0.15 m^3^ air per m^2^ membrane per h, or 10 m^3^ air per m^3^ permeate) for membrane fouling control by a blower. The two pilot-scale MBRs were operated for a total of 74 d with 4 stages according to different influent loads and average membrane flux. The operation parameters and influent quality during the 4 stages of the pilot-scale MBRs in this study are shown in Table 1 and Table 2, respectively. The MBR pilot test was conducted in 4 stages in sequences of low, medium, high, and over-load corresponding to pumping activated sludge mixed liquor from the terminal, middle, and initial (for both high and over-load) zones of the aeration tank into the membrane tank, with an average membrane flux of 15, 15, 15, and 18/22.5 L/(m^2^·h), respectively, resulting in the MBR pilot treatment capacities with the PVDF and PES membranes of 151.2–226.8 m^3^/d and 169.3–253.8 m^3^/d. The influent, the mixed liquor in the membrane tank, and the effluent were sampled daily to monitor membrane rejection performance, while TMP was recorded in real-time to monitor membrane fouling development. In addition, after 74 d of operation, the new and fouled membranes were sampled for the characterization of pure water permeability, pore size distribution, contact angle, and surface zeta potential to reflect membrane changes.

### 2.3. Analytical Methods

The water quality during the experiment was analyzed according to the routine standards [23]: COD was determined by the oxidation of potassium dichromate followed by titration with ferrous ammonium sulfate; BOD was measured via the five-day dilution incubation method; TN was determined by alkaline potassium persulfate digestion followed by UV spectrophotometry; chromaticity was determined by the dilution visual colorimetric method; turbidity was measured via an infrared scattering turbidimeter; and MLSS was detected by 0.45 μm glass fiber filtration followed by heating. The samples from influent and mixed liquor in the membrane tanks were pre-filtered via a 0.45 μm glass fiber filter for soluble fraction measurement, except for MLSS. Membrane rejection performance R is defined as R = (1 − C_eff_/C_tank_) × 100%, where C_eff_ and C_tank_ are pollutant concentrations in the membrane effluent and the membrane tank, respectively.

Both new and fouled membranes, after 74 d operation, were characterized by pure water permeability, pore size distribution, contact angle, and surface zeta potential. Pure water permeability was detected by pure water dead-end filtration at a constant TMP of 100 kPa and a temperature of 20 °C. Pore size distribution was detected via the bubbling method, where the gas flow, through isopropanol (surface tension of 21.7 mN/m at 25 °C) wetted then followed by a dry membrane, and the pressure were measured by a porosity meter [24]. Contact angle was detected by the high-speed video recording method, where a 5 µL water droplet was dropped on the membrane surface, and a static image was captured by a contact angle analyzer [25]. Surface zeta potential was detected by a streaming potential current analyzer with 1 mmol/L KCl solution (pH of 3–10, temperature of 25 °C) as the test solution and a 20 × 10 mm membrane cell [26].

## 3. Results and Discussion

### 3.1. Rejection Capability of Two Flat-Sheet Microfiltration Membranes in Pilot-Scale MBRs

#### 3.1.1. Organics Rejection

**Figure 2:**
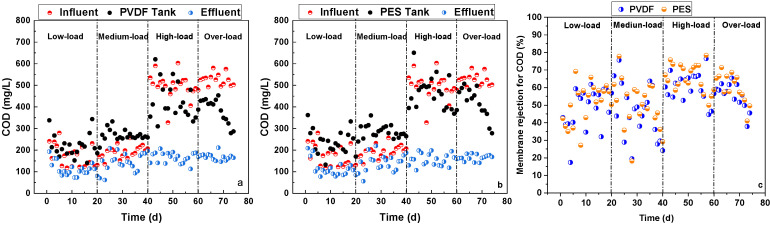
COD changes in pilot-scale MBRs with PVDF membrane (**a**) and PES membrane (**b**), and COD rejection ratios of PVDF and PES membranes (**c**).

The COD rejection performance of the two flat-sheet microfiltration membranes in the pilot-scale MBRs is shown in Figure 2. During the low- and medium-load stages, the CODs of the activated sludge mixed liquor in the membrane tanks were mostly higher than that of the influent (i.e., activated sludge mixed liquor in the terminal and middle zone of aeration tank after 8–16 h aerobic biodegradation in the textile dyeing WWTP). This could be derived from the concentrating effects of both sludge and macromolecular organics by the microfiltration membrane [27]. The MLSS in the membrane tanks was around 12 g/L, significantly higher than the influent MLSS of around 3 g/L. The high MLSS in the membrane tanks enhanced endogenous respiration and hydrolysis/degradation of microbial cells, thus releasing more soluble microbial products as COD contributors. The direct rejection of macromolecular organics by microfiltration membranes also increased the COD in the membrane tanks. However, during the high- and over-load stages, the CODs of the activated sludge mixed liquor in membrane tanks were mostly lower than that of the influent (i.e., activated sludge mixed liquor in the initial zone of aeration tank with minimal biodegradation in the textile dyeing WWTP). This could be derived from the high-concentration biodegradable COD in the influent, which was mostly biodegraded in the membrane tanks with a hydraulic rejection time of around 2 h. During all four stages, the membrane effluent COD was always lower than the COD in the influent and the membrane tanks due to the direct rejection of macromolecular organics by the microfiltration membranes. During the low-, medium-, high-, and over-load stages, as well as for the whole experiment, the PVDF and PES membranes achieved average COD rejection ratios of 48.3% and 51.4%, 45.7% and 50.1%, 60.4% and 65.8%, 55.5% and 59.9%, and 52.5% and 56.8%, respectively, demonstrating agreement with previous studies [27,28]. In summary, the PES membrane showed slightly better COD rejection performance than the PVDF membrane.

**Figure 3:**
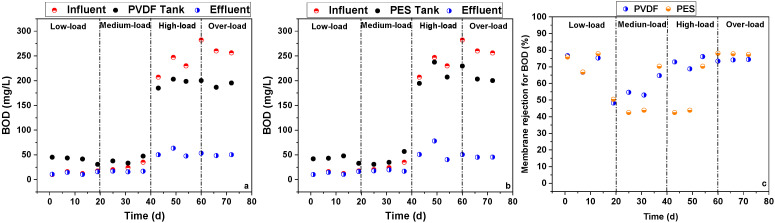
BOD changes in pilot-scale MBRs with PVDF membrane (**a**) and PES membrane (**b**), and BOD rejection ratios of PVDF and PES membranes (**c**).

The BOD rejection performance of the two flat-sheet microfiltration membranes in the pilot-scale MBRs is shown in Figure 3. Similarly to the COD trend, during the low- and medium-load stages, the BOD of the activated sludge mixed liquor in the membrane tanks was higher and lower than that of the influent between the low-/medium-load and high-/over-load stages, respectively. During all four stages, the membrane effluent BOD was always lower than the BOD in the influent and the membrane tanks due to the direct rejection of macromolecular organics by the microfiltration membranes. During the low-, medium-, high-, and over-load stages, as well as for the whole experiment, the PVDF and PES membranes achieved average COD rejection ratios of 66.7% and 67.8%, 57.5% and 52.2%, 72.8% and 58.7%, 74.3% and 77.6%, and 67.8% and 64.1%, respectively. In summary, the PVDF membrane showed slightly better BOD rejection performance than the PES membrane.

**Figure 4:**
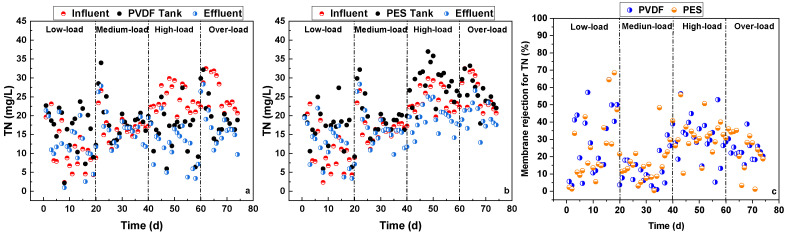
TN changes in pilot-scale MBRs with PVDF membrane (**a**) and PES membrane (**b**), and TN rejection ratios of PVDF and PES membranes (**c**).

The TN rejection performance of the two flat-sheet microfiltration membranes in the pilot-scale MBRs is shown in Figure 4. During all four stages, the TN of the activated sludge mixed liquor in the membrane tanks was higher than that of the influent, which can be attributed to the above-mentioned concentrating effects by both the membrane and the very limited TN assimilation without denitrification under the fully aerobic conditions in both membrane tanks. This accumulated TN in the membrane tank, and limited TN rejection by both membranes, even resulting in a higher TN in membrane effluent than that of the influent during the low- and medium-load stages. During all four stages, the membrane effluent TN was always lower than the TN in the membrane tanks due to the direct rejection of macromolecular organics containing nitrogen by the microfiltration membranes. During the low-, medium-, high-, and over-load stages, as well as for the whole experiment, the PVDF and PES membranes achieved average TN rejection ratios of 27.9% and 24.8%, 13.2% and 16.5%, 30.9% and 31.6%, 23.6% and 23.5%, and 23.9% and 24.1%, respectively. In summary, the PVDF membrane showed nearly the same TN rejection performance as the PES membrane.

#### 3.1.2. Turbidity and Chromaticity Rejection

**Figure 5:**
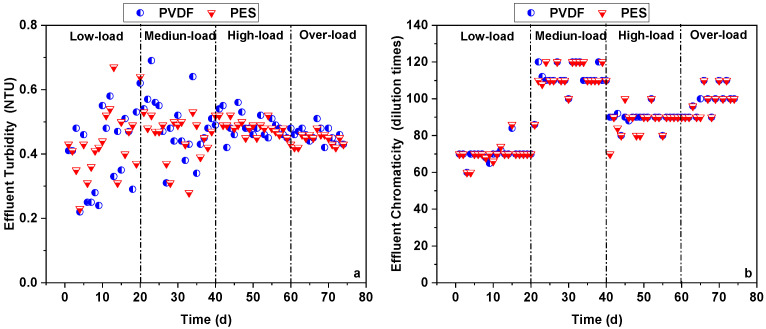
Effluent turbidity (**a**) and chromaticity (**b**) in pilot-scale MBRs.

The effluent turbidity of the two flat-sheet microfiltration membranes in the pilot-scale MBRs is shown in Figure 5a. During the low-load stage, the effluent turbidity of the PVDF and PES membranes fluctuated, ranging 0.25–0.58 NTU and 0.23–0.67 NTU, respectively, with averages of 0.40 NTU and 0.42 NTU. During the medium-load stage, the effluent turbidity of the PVDF and PES membranes fluctuated, ranging 0.31–0.69 NTU and 0.28–0.53 NTU, respectively, with averages of 0.49 NTU and 0.46 NTU. During the high-load stage, the effluent turbidity of the PVDF and PES membranes fluctuated, ranging 0.42–0.56 NTU and 0.43–0.52 NTU, respectively, with averages of 0.49 NTU and 0.48 NTU. During the over-load stage, the effluent turbidity of the PVDF and PES membranes fluctuated, ranging 0.42–0.51 NTU and 0.42–0.58 NTU, respectively, with averages of 0.46 NTU and 0.45 NTU. In summary, both the PVDF and PES membranes showed nearly the same effluent turbidity, which could be derived from the effective rejection of turbidity substances (i.e., suspended particles and colloids) by both membranes.

The effluent chromaticity of the two flat-sheet microfiltration membranes in the pilot-scale MBRs is shown in Figure 5b. During the low-load stage, the effluent chromaticity of the PVDF and PES membranes fluctuated 60–84 times and 60–86 times, respectively, with both averaging around 70 times. During the medium-load stage, the effluent chromaticity of both the PVDF and PES membranes fluctuated 86–120 times, with an average of around 110 times. During the high-load stage, the effluent chromaticity of the PVDF and PES membranes fluctuated 80–100 times and 70–100 times, respectively, with both averaging around 90 times. During the over-load stage, the effluent chromaticity of both the PVDF and PES membranes fluctuated 90–110 times, with an average of around 100 times. The effluent chromaticity in this study was similar to that from a previous study [29]. In summary, both the PVDF and PES membranes showed nearly the same effluent chromaticity, which could be derived from the ineffective rejection of colored substances (i.e., soluble humics) by both membranes.

### 3.2. Antifouling Capability of Two Flat-Sheet Microfiltration Membranes in Pilot-Scale MBRs

#### 3.2.1. Membrane Fouling Development in MBRs

**Figure 6:**
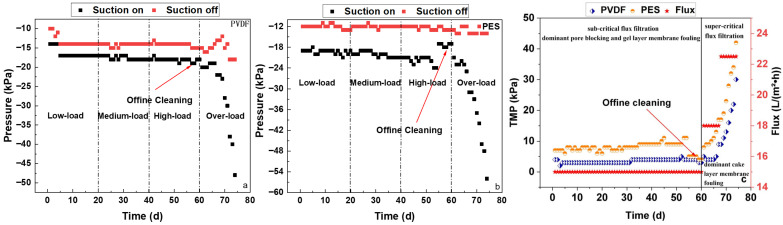
Daily average pressure (**a**,**b**) and TMP (**c**) of PVDF/PES membrane in pilot-scale MBRs.

The daily average pressure, with and without suction permeating, of the PVDF and PES membranes in the pilot-scale MBRs is shown in Figure 6a and Figure 6b, respectively. The average negative pressures without suction permeating were 10–18 kPa and 10–15 kPa for the PVDF and PES membranes, respectively, which were mainly affected by the water level fluctuation in the membrane tanks. The average negative pressure with suction permeating was 14–48 kPa and 16–57 kPa for the PVDF and PES membranes, respectively, which was mainly affected by membrane resistance caused by membrane fouling.

As shown in Figure 6c, there were stable TMPs of around 3 kPa and 7 kPa with negligible increases below 0.05 kPa/d during both the low- and medium-load stages, with a long-time filtration of 20 d and an average flux of 15 L/(m^2^·h) for the PVDF and PES membranes, respectively. This demonstrates the typical characteristics of sub-critical flux filtration with the occurrence of low-resistance pore blocking and gel layer fouling, mainly caused by organics (e.g., soluble microbial products) [30,31,32]. This indicated no obvious fouling development for both membranes, which could be derived from the low soluble COD in the membrane tanks (shown in Figure 2), which is mainly caused by the low influent soluble COD after finishing aerobic biodegradation. During the high-load stage, with an average flux of 15 L/(m^2^·h), the TMP of the PVDF membrane still stabilized, with an approximate 4 kPa negligible increase below 0.05 kPa/d, while the TMP of the PES membrane gradually increased from 9 kPa at 41–44 d to 10–12 kPa at 45–54 d, with an average of 0.15 kPa/d, indicating significant pore blocking and cake layer fouling development, possibly due to the stronger interaction between the membrane material and the foulants [33]. Considering the similar soluble COD in both membrane tanks, the different membrane fouling behaviors could be derived from the different membrane characteristics discussed below. Offline chemical cleaning via emptying the membrane tanks, followed by filling them with sodium hypochlorite solution (active chlorine of 3000 mg/L, pH of 12, and ambient temperature of 15–20 °C) for membrane soaking spanning 12 h was thus conducted for both PVDF and PES membranes before the over-load stage, in order to obtain a better comparison in this study. The initial TMPs of the PVDF and PES membranes during the over-load stage were around 3 kPa and 4 kPa, respectively. During the following 1 week (i.e., 61–67 d), with an average flux of 18 L/(m^2^·h), the TMP of the PVDF membrane increased to 9 kPa with an average TMP increase rate of 0.86 kPa/d, while the TMP of the PES membrane increased to 18 kPa, with an average TMP increase rate of 2 kPa/d, both showing the transition from sub-critical flux operation without cake layer fouling to super-critical flux with dominant cake layer fouling [30,31,32]. From 68 to 74 d, with an average flux of 22.5 L/(m^2^·h), the TMP of the PVDF membrane further increased to 30 kPa, with an average TMP increase rate of 3 kPa/d, while the TMP of the PES membrane further increased to 42 kPa, with an average TMP increase rate of 3.4 kPa/d, both showing the typical characteristics of super-critical flux filtration with dominant cake layer fouling, caused by sludge flocs deposition [30,31,32]. In summary, the PVDF membrane showed a lower TMP increase rate and thus better anti-fouling performance than the PES membrane during the high- and over-load stages.

#### 3.2.2. Characteristics of New and Fouled Membranes

The pure water permeability and pore size distribution of the new and fouled flat-sheet membranes are shown in Table 3. The pure water permeability of the new PVDF membrane was 242 m^3^/(m^2^·h·MPa), which was 22.6 times as much as 10.7 m^3^/(m^2^·h·MPa) from the new PES membrane, and also, the highest one for an MBR application, to the best of our knowledge. The average pore size of the new PVDF membrane was 2.13 μm, which was 11.3 times as much as 0.188 μm from the new PES membrane. The maximum pore size of the new PVDF membrane was 3.15 μm, which was 10.5 times as much as 0.3 μm from the new PES membrane, while the bubble point pressure of the new PES membrane was 0.2 MPa, which was 10.5 times as much as 0.019 MPa from the new PVDF membrane, both showing good agreement. The much larger pore size of the new PVDF membrane than that of the new PES membrane was the dominant reason behind the much larger pure water permeability of the new PVDF membrane than the new PES membrane. After 74 d of filtration in the pilot-scale MBRs, the pure water permeability of the fouled PVDF membrane was 45.7 m^3^/(m^2^·h·MPa) and it only retained 18.9% of the new PVDF membrane, while the pure water permeability of the fouled PES membrane was 10 m^3^/(m^2^·h·MPa) and still retained 93.5% of new PES membrane. This indicated much stronger effects of residual fouling on pure water permeability in the fouled PVDF membrane than in the fouled PES membrane. The fouled PVDF membrane had a significantly smaller average pore size of 1.35 μm than the new PVDF membrane, while the fouled PVDF membrane had a slightly larger maximum pore size of 3.33 μm and a corresponding slightly lower bubble point pressure of 0.018 MPa. Both showed different trends, which might be due to the distribution heterogeneity of both membrane pore size and fouling [34]. The fouled PES membrane had a significantly smaller average pore size of 0.1 μm, a significantly smaller maximum pore size of 0.133 μm, and a significantly higher bubble point pressure of 0.45 MPa than those of the new PES membrane, showing the same trend. Although a larger permeability loss for the PVDF membrane than the PES membrane, the residual permeability of the fouled PVDF membrane was still much higher than that of the fouled PES membrane, thus leading to the lower TMP of the PVDF membrane than the PES membrane during the pilot-scale MBR experiments (shown in Figure 6c).

Contact angle and surface potential of the new and fouled flat-sheet membranes are shown in Table 4. The contact angles of the new PVDF and PES membranes were measured as 46° and 66.7°, respectively, showing a more hydrophilic surface in the new PVDF membrane than the new PES membrane. The contact angle of the fouled PVDF membrane sample, after the 74 d plot test, showed a significant increase to 81.4°, which could be derived from the loss of hydrophilic pore-forming agents or additives in the membrane caused by the continuous filtration and the offline chemical cleaning [35]. The contact angle of the fouled PES membrane, after the 74 d pilot test, showed a slight decrease to 61°, which could be attributed to the chemical modification effect of the NaClO solution in the offline chemical cleaning solution on the polymer surface, enhancing the hydrophilicity of the membrane surface [36]. The surface potentials of the new PVDF and PES membranes were measured at −64.6 mV and −41.4 mV, respectively, showing a more negatively charged surface in the new PVDF membrane than in the new PES membrane. After the 74 d pilot test, there was a significant surface potential decrease to −96.9 mV and −73.5 mV for the fouled PVDF and PES membranes, respectively, indicating more negative groups enriched during the filtration. For the PVDF membrane, the hydroxyl content increase might be the reason for the negative charge enrichment on the membrane surface [37]. For the PES membrane, the sulfonic acid groups might have increased the number of ionizable groups on the membrane surface and thus enhanced the net surface charge density [38].

### 3.3. Implication for Membrane Selection in MBR Retrofit of Textile Dyeing WWTP

Based on the above-mentioned results from the pilot-scale MBRs with both membranes, the PVDF membrane had nearly the same rejection capability as and better anti-fouling capability (especially during high-/over-load stages) than the PES membrane. Therefore, the PVDF membrane was suggested for the MBR retrofit of the textile dyeing WWTP. The pore size of PVDF membrane was significantly larger than that of a normal MF (0.1–0.4 µm) used in conventional MBRs but significantly smaller than the microscreen substrate (20–100 µm) used in dynamic MBRs [39], which could be the reason for its higher permeability than and similar rejection capability to a normal MF via both pore blocking and gel layer controlled separation. Although the PVDF membrane could effectively meet the over-load challenges in a short time, it is still suggested to place the membrane modules in the terminal zone of the aeration tank (i.e., filtrating activated sludge mixed liquor after finishing the biodegradation of raw wastewater). We further suggest maintaining a sustainable membrane flux below 18 L/(m^2^·h) and keeping a maximum MLSS below 15,000 mg/L to achieve optimal performance, which includes membrane effluent quality, fouling control, and volumetric loading rate, similarly to the general MBR practice for municipal wastewater treatment [12,40]. In addition, the optimal cleaning strategy for the PVDF membrane might be different from both conventional and dynamic MBRs and needs further study.

The significant difference in membrane pore size distribution and material, yet keeping nearly the same rejection and anti-fouling capability during the low-/medium-load stages of the pilot-scale MBR experiments between the PVDF and PES membranes, was identified in this study, which was different from other previous MBR studies [41,42,43]. This might be derived from the specific interactions between the membrane and foulant [33], minimizing the pristine effects of both membranes in this specific MBR for textile dyeing wastewater treatment. Compared with the PES membrane, the PVDF membrane, with its significantly larger pore size distribution, could produce some self-adaptive changes (e.g., enhanced adsorption and electrostatic repulsion for improving rejection capability) in this specific wastewater matrix, and thus, function with nearly the same rejection capability [44]. This was also reflected by more characteristic changes observed between the new and fouled membranes of PVDF than those of the PES membrane.

## 4. Conclusions

Two commercial flat-sheet microfiltration membranes made of PVDF and PES material were systematically evaluated in terms of rejection and anti-fouling capability, side by side in two parallel pilot-scale MBRs with a treatment capacity of 150–250 m^3^/d for the in situ retrofitting of a textile dyeing WWTP. Each MBR pilot test was conducted in four stages, in a sequence of low, medium, high, and over-load corresponding to the pumping of activated sludge mixed liquor from the terminal, middle, and initial (for both high and over-load) zones of an aeration tank into a membrane tank, with average membrane flux of 15, 15, 15, and 18/22.5 L/(m^2^·h), respectively. During the whole operation, the PVDF and PES membranes achieved similar organics rejection in terms of COD (52.5% and 56.8%), BOD (67.8% and 64.1%), and TN (23.9% and 24.1%), respectively, and produced similar effluent chromaticity (60–120 dilution times) and turbidity (0.2–0.7 NTU). Both the PVDF and PES membranes showed little TMP increase below 0.15 kPa/d, at an average flux of 15 L/(m^2^·h) with sub-critical flux filtration characteristics. Both the PVDF and PES membranes showed a sharp TMP increase with 0.86 and 2 kPa/d at an average flux of 18 L/(m^2^·h) and with 3 and 3.4 kPa/d at an average flux of 22.5 L/(m^2^·h), respectively, indicating super-critical flux filtration characteristics. After 74 d filtration and at an average sludge concentration of 12,000 mg/L, the PVDF membrane showed less variation in pore size distribution and bubble point pressure, while the PES membrane showed less change in permeability and contact angle. Despite significant differences in membrane pore size distribution and material, both the PVDF and PES membranes met general MBR requirements, which was mainly due to minimizing the pristine effects of both membranes by this specific textile dyeing wastewater matrix. The PVDF membrane showed better anti-fouling capability, especially during high-/over-load stages due to its larger pore size distribution and pure water permeability, and thus, was suggested for this MBR retrofit. It was also suggested to place membrane modules in the terminal zone of the aeration tank (i.e., filtrating activated sludge mixed liquor after finishing biodegradation of raw wastewater), maintain a sustainable membrane flux below 18 L/(m^2^·h), and keep a maximum MLSS below 15,000 mg/L for achieving optimal performance, including membrane effluent quality, fouling control, and volumetric loading rate.

## Figures and Tables

**Table 1:** Operation parameters of two parallel pilot-scale MBRs.

Stage	Time	Influent	Average Flux
Low load	1–20 d	Activated sludge mixed liquor from terminal zone of aeration tank in WWTP	15 L/(m^2^·h)
Medium load	21–40 d	Activated sludge mixed liquor from middle zone of aeration tank in WWTP	15 L/(m^2^·h)
High load	41–60 d	Activated sludge mixed liquor from initial zone of aeration tank in WWTP	15 L/(m^2^·h)
Over-load	61–67 d 68–74 d	Activated sludge mixed liquor from initial zone of aeration tank in WWTP	18 L/(m^2^·h) 22.5 L/(m^2^·h)

**Table 2:** The influent quality of the two parallel pilot-scale MBRs.

Stage	Time	Soluble COD (mg/L)	Soluble BOD (mg/L)	Soluble TN (mg/L)	Soluble Chromaticity (Dilution Times)	MLSS (g/L)
Low load	1–20 d	123–280	12–20	2.3–23.1	80–100	2.2–3.3
Medium load	21–40 d	135–257	20–35	11.1–26.6	120–200	2.1–3.7
High and over-load	41–74 d	327–603	207–282	19.7–23.5	200–400	1.9–3.6

**Table 3:** Pure water permeability and pore size distribution of new and fouled membranes.

Membrane Sample	Pure Water Permeability [m^3^/(m^2^·h·MPa)]	Average Pore Size (μm)	Bubble Point Pressure (MPa)	Maximum Pore Size (μm)
PVDF new membrane	242	2.13	0.019	3.15
PES new membrane	10.7	0.188	0.20	0.30
PVDF fouled membrane	45.7	1.35	0.018	3.33
PES fouled membrane	10.0	0.10	0.45	0.133

**Table 4:** Contact angle and surface potential of new and fouled membranes.

Membrane Sample	Contact Angle (°)	Surface Potential (mV)
PVDF new membrane	46.0	−64.6
PES new membrane	66.7	−41.4
PVDF fouled membrane	81.4	−96.9
PES fouled membrane	61.0	−73.5

## Data Availability

The original contributions presented in this study are included in the article. Further inquiries can be directed to the corresponding authors.

## References

[1] Ministry of Ecology and Environment of the People’s Republic of China (2025). China Environmental Statistics Yearbook 2024.

[2] Dong D.P., Wu M.Y., Yang Y.F., Jiao Y.S., Wang B., Shao M., Zhao X.Z. (2025). Textile Printing and Dyeing Wastewater: A Comprehensive Profiling and In-Depth Examination of Sustainable Treatment Strategies. ChemistrySelect.

[3] Kishor R., Purchase D., Saratale G.D., Saratale R.G., Ferreira L.F.R., Bilal M., Chandra R., Bharagava R.N. (2021). Ecotoxicological and health concerns of persistent coloring pollutants of textile industry wastewater and treatment approaches for environmental safety. J. Environ. Chem. Eng..

[4] Lin W.J., Lin W., Yang L.Z., Zhang C.F., Li X.H., Xiao J.B., Chen X.T., Cai F., Chen C., Zhang M.L. (2026). Characterization of a novel Dye-Decolorizing peroxidase from Brevibacillus agri and its application in detoxification of textile industry wastewater. Bioresour. Technol..

[5] Liu Z.C., Khan T.A., Islam M.A., Tabrez U. (2022). A review on the treatment of dyes in printing and dyeing wastewater by plant biomass carbon. Bioresour. Technol..

[6] Zhou C.Q., Gu H.K., Wei C.H., Rong H.W., Ng H.Y. (2022). Dyeing and finishing wastewater treatment via a low-cost hybrid process of hydrolysis-acidification and alternately anoxic/oxic sequencing batch reactor with synchronous coagulation. J. Water Process Eng..

[7] Bogale F.M., Teffera B., Aragaw T.A. (2024). Recent developments in integrated anaerobic/aerobic (A/O) process for textile industry wastewater treatment: A review. J. Hazard. Mater. Adv..

[8] Chen H., Yu X., Wang X.N., He Y.L., Zhang C.J., Xue G., Liu Z.H., Lao H.B., Song H.L., Chen W. (2021). Dyeing and finishing wastewater treatment in China: State of the art and perspective. J. Clean. Prod..

[9] Cinperi N.C., Ozturk E., Yigit N.O., Kitis M. (2019). Treatment of woolen textile wastewater using membrane bioreactor, nanofiltration and reverse osmosis for reuse in production processes. J. Clean. Prod..

[10] Xu H.B., Zhang H., Huang J.C., Zhang L., Wang F., Liu G.B., Yu X.M., Liu W.J., Huang C.K. (2025). Optimization of Fenton combined with membrane bioreactor in the treatment of printing and dyeing wastewater. Int. Biodeterior. Biodegrad..

[11] Saraiva B., Belino N., Miguel R., Lopez-Grimau V., Buscio V., Carvalho J., Fernandes A. (2025). Treatment of natural dye wastewaters by electrochemical oxidation: Comparative analysis of anode materials and optimization for reuse. J. Environ. Manag..

[12] Judd C., Judd S. The MBR Site. https://www.thembrsite.com/.

[13] Zhang D.Q., Trzcinski A.P., Kunacheva C., Stuckey D.C., Liu Y., Tan S.K., Ng W.J. (2016). Characterization of soluble microbial products (SMPs) in a membrane bioreactor (MBR) treating synthetic wastewater containing pharmaceutical compounds. Water Res..

[14] Guo H., Xu W.C., Gao Y.H., Duan F., Zhang Y.X., Cao H.B. (2026). Treatment of spent lithium-ion battery discharging wastewater by MBR under increasing salinity: Performance, membrane fouling, and microbial community succession. J. Water Process Eng..

[15] Chen F., Ma J., Zhu Y.F., Li X.X., Yu H.C., Sun Y. (2022). Biodegradation performance and anti-fouling mechanism of an ICME/electro-biocarriers-MBR system in livestock wastewater (antibiotic-containing) treatment. J. Hazard. Mater..

[16] Zhang R., Xin B.Y., Zhang C., Ma X.Z., Wang R.X., Guo J.F. (2026). Targeted interception of membrane foulants in MBR: A straw-microplastic composite adsorbent for effective removal of oil and biopolymeric contaminants from catering wastewater. J. Hazard. Mater..

[17] Gao T.W., Xiao K., Zhang J., Xue W.C., Wei C.H., Zhang X.P., Liang S., Wang X.M., Huang X. (2022). Techno-economic characteristics of wastewater treatment plants retrofitted from the conventional activated sludge process to the membrane bioreactor process. Front. Environ. Sci. Eng..

[18] Praneeth K., Moulik S., Vadthya P., Bhargava S.K., Tardio J., Sridhar S. (2014). Performance assessment and hydrodynamic analysis of a submerged membrane bioreactor for treating dairy industrial effluent. J. Hazard. Mater..

[19] Moazzem S., Ravishankar H., Fan L.H., Roddick F., Jegatheesan V. (2020). Application of enhanced membrane bioreactor (eMBR) for the reuse of carwash wastewater. J. Environ. Manag..

[20] Bilici Z., Unal B.O., Ozay Y., Keskinler B., Karagunduz A., Orhon D., Dizge N. (2020). Effluent reuse potential of a dual-stage ceramic MBR coupled with RO treatment for textile wastewater. Desalination Water Treat..

[21] Feng F., Xu Z.L., Li X.H., You W.T., Zhen Y. (2010). Advanced treatment of dyeing wastewater towards reuse by the combined Fenton oxidation and membrane bioreactor process. J. Environ. Sci..

[22] (2012). Discharge Standards of Water Pollutants for Dyeing and Finishing of Textile Industry.

[23] APHA, AWWA, WEF (2017). 1995 Standard Methods for the Examination of Water and Wastewater.

[24] Yang Y.B., Chai W.Q., Zhang L., Wang J.Y., You J.C. (2024). A mini-review of polymeric porous membranes with vertically penetrative pores. J. Polym. Sci..

[25] Arumugham T., Kaleekkal N.J., Gopal S., Nambikkattu J., Rambabu K., Aboulella A.M., Wickramasinghe S.R., Banat F. (2021). Recent developments in porous ceramic membranes for wastewater treatment and desalination: A review. J. Environ. Manag..

[26] Disalvo A., Frias M.A. (2021). Surface Characterization of Lipid Biomimetic Systems. Membranes.

[27] Du X., Shi Y., Jegatheesan V., Ul Haq I. (2020). A Review on the Mechanism, Impacts and Control Methods of Membrane Fouling in MBR System. Membranes.

[28] Samsami S., Mohamadi M., Sarrafzadeh M.-H., Rene E.R., Firoozbahr M. (2020). Recent advances in the treatment of dye-containing wastewater from textile industries: Overview and perspectives. Process Saf. Environ. Prot..

[29] Wei C., Lao Y., Ouyang R., Zhang G., Huang G., Deng F., Tan Q., Lin G., Zhou H. (2023). Evaluation of Different Reverse Osmosis Membranes for Textile Dyeing and Finishing Wastewater Reuse. Membranes.

[30] Chu H., Zhang Y., Zhou X., Zhao Y., Dong B., Zhang H. (2014). Dynamic membrane bioreactor for wastewater treatment: Operation, critical flux, and dynamic membrane structure. J. Membr. Sci..

[31] Wei C.-H., Huang X., Ben Aim R., Yamamoto K., Amy G. (2011). Critical flux and chemical cleaning-in-place during the long-term operation of a pilot-scale submerged membrane bioreactor for municipal wastewater treatment. Water Res..

[32] Xu R.L., Fan Y.B., Yang M., Song J.Q. (2023). Determination of Sustainable Critical Flux through a Long-Term Membrane Resistance Model. Polymers.

[33] Xu H., Xiao K., Wang X., Liang S., Wei C., Wen X., Huang X. (2020). Outlining the Roles of Membrane-Foulant and Foulant-Foulant Interactions in Organic Fouling During Microfiltration and Ultrafiltration: A Mini-Review. Front. Chem..

[34] Guo Y., Li C., Zhao H., Wang X., Gao M., Sun X., Wang Q. (2023). The Performance of Ultrafiltration Process to Further Refine Lactic Acid from the Pre-Microfiltered Broth of Kitchen Waste Fermentation. Membranes.

[35] Lv J., Zhang G., Zhang H., Yang F. (2018). Graphene oxide-cellulose nanocrystal (GO-CNC) composite functionalized PVDF membrane with improved antifouling performance in MBR: Behavior and mechanism. Chem. Eng. J..

[36] Ghalamchi L., Aber S., Vatanpour V., Kian M. (2019). A novel antibacterial mixed matrixed PES membrane fabricated from embedding aminated Ag_3_PO_4_g-C_3_N_4_ nanocomposite for use in the membrane bioreactor. J. Ind. Eng. Chem..

[37] Liu J., Xiong J., Ju X., Gao B., Wang L., Sillanpaa M. (2018). Streaming potential for identification of foulants adsorption on PVDF membrane surface. J. Membr. Sci..

[38] Yu H., Shangguan S., Xie C., Yang H., Wei C., Rong H., Qu F. (2022). Chemical Cleaning and Membrane Aging in MBR for Textile Wastewater Treatment. Membranes.

[39] Mohan S.M., Nagalakshmi S. (2020). A review on aerobic self-forming dynamic membrane bioreactor: Formation, performance, fouling and cleaning. J. Water Process Eng..

[40] (2022). Technical Specification for In-Situ Retrofit of Municipal Wastewater Treatment Plants Based on Membrane Bioreactor Processes.

[41] Zhu T., Xie Y.H., Jiang J., Wang Y.T., Zhang H.J., Nozaki T. (2009). Comparative study of polyvinylidene fluoride and PES flat membranes in submerged MBRs to treat domestic wastewater. Water Sci. Technol..

[42] Jeon S., Rajabzadeh S., Okamura R., Ishigami T., Hasegawa S., Kato N., Matsuyama H. (2016). The Effect of Membrane Material and Surface Pore Size on the Fouling Properties of Submerged Membranes. Water.

[43] Raja W., Kumar P. (2025). Membrane bioreactors for wastewater treatment: Advances, fouling control strategies, and integration into the circular economy. Indian Chem. Eng..

[44] Maiti S., Islam S.S., Bose S. (2025). Nano-Engineering for Purity: Advances in PVDF Membrane Water Purification. Chem. Asian J..

